# *Akkermansia muciniphila* inversely correlates with the onset of inflammation, altered adipose tissue metabolism and metabolic disorders during obesity in mice

**DOI:** 10.1038/srep16643

**Published:** 2015-11-13

**Authors:** Marc Schneeberger, Amandine Everard, Alicia G. Gómez-Valadés, Sébastien Matamoros, Sara Ramírez, Nathalie M. Delzenne, Ramon Gomis, Marc Claret, Patrice D. Cani

**Affiliations:** 1 Diabetes and Obesity Research Laboratory, Institut d’Investigacions Biomèdiques August Pi i Sunyer (IDIBAPS), 08036 Barcelona, Spain; 2 Department of Endocrinology and Nutrition, Hospital Clínic. School of Medicine, University of Barcelona, 08036 Barcelona, Spain; 3 CIBER de Diabetes y Enfermedades Metabólicas Asociadas (CIBERDEM), 08036 Barcelona, Spain; 4 Université catholique de Louvain, Louvain Drug Research Institute, WELBIO- Walloon Excellence in Life Sciences and BIOtechnology, Metabolism and Nutrition Research Group, Brussels, Belgium

## Abstract

Recent evidence indicates that the gut microbiota plays a key role in the pathophysiology of obesity. Indeed, diet-induced obesity (DIO) has been associated to substantial changes in gut microbiota composition in rodent models. In the context of obesity, enhanced adiposity is accompanied by low-grade inflammation of this tissue but the exact link with gut microbial community remains unknown. In this report, we studied the consequences of high-fat diet (HFD) administration on metabolic parameters and gut microbiota composition over different periods of time. We found that *Akkermansia muciniphila* abundance was strongly and negatively affected by age and HFD feeding and to a lower extend *Bilophila wadsworthia* was the only taxa following an opposite trend. Different approaches, including multifactorial analysis, showed that these changes in *Akkermansia muciniphila* were robustly correlated with the expression of lipid metabolism and inflammation markers in adipose tissue, as well as several circulating parameters (i.e., glucose, insulin, triglycerides, leptin) from DIO mice. Thus, our data shows the existence of a link between gut *Akkermansia muciniphila* abundance and adipose tissue homeostasis on the onset of obesity, thus reinforcing the beneficial role of this bacterium on metabolism.

The digestive tract is colonized by trillions of microbes (i.e., the gut microbiota) that exert a wide range of beneficial functions for the host. In recent years, it has become apparent that alterations in gut microbiota composition is associated with the development of highly prevalent metabolic disorders such as obesity and type 2 diabetes (T2D) in both animal and human studies^1^,^2^,^3^,^4^,^5^,^6^,^7^. We and others have demonstrated that high-fat diet (HFD) feeding profoundly affects the gut microbial community^1^,^8^,^9^,^10^,^11^,^12^,^13^,^14^. In particular, the abundance of *Bifidobacterium* spp. and *Akkermansia muciniphila* is consistently reduced under HFD regimen^8^,^14^,^15^. We also observed a decrease in *Lactobacillus* spp. and *Bacteroides/Prevotella* spp. However, the reduced abundance of these genera was less consistent^16^,^17^,^18^ suggesting that these changes were not involved in the development of the disorders associated with diet-induced obesity (DIO). Recently, increased abundance of *Bilophila wadsworthia* has been associated with fat feeding and inflammation^7^,^19^.

The pathological massive expansion of adipose tissue in obesity states is associated with the development of low-grade inflammation, which is reflected by enhanced production of cytokines, chemokines and pro-inflammatory fatty acids. This causes an imbalance between pro- and anti-inflammatory factors produced by leukocytes, further promoting inflammation and adipose tissue dysfunction (e.g., β-oxidation, browning processes, adipogenesis)^20^,^21^,^22^,^23^. Despite the well-established link between DIO and gut microbiota alterations, it is currently unknown whether changes in the abundance of specific bacteria precedes or remains present during the development of obesity and related adipose tissue metabolic alterations.

In the current study we explored the potential interconnection between adipose tissue inflammation/function and changes in specific microbes during obesity and T2D development following chronic HFD feeding in mice. Our results demonstrate specific changes in gut bacteria over the progress of obesity, together with a robust negative association between *Akkermansia muciniphila* and inflammation and a positive association with adipose tissue browning process markers. This work also demonstrates that this bacterium declines before the onset of metabolic alterations, thereby suggesting a putative causative implication in the disease progression.

## Results

### Body weight and adiposity progression over 16 weeks of HFD feeding

**Figure 1:**
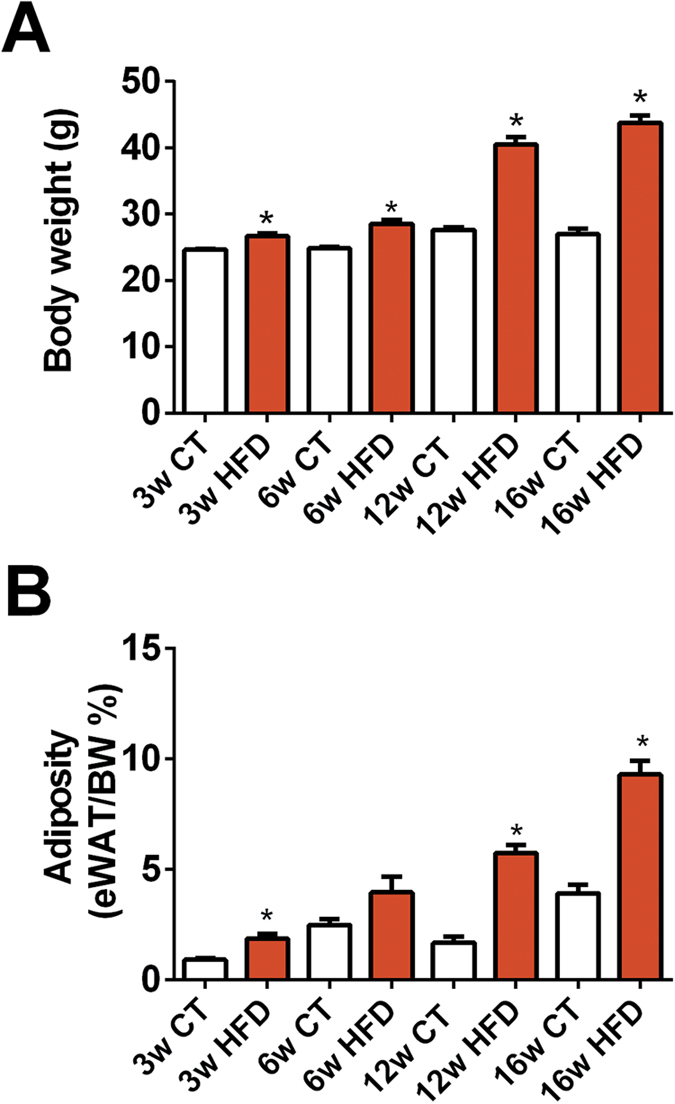
Kinetic evolution of body weight and adiposity following chronic HFD administration. (**A**) Body weight gain (g) and (**B**) adiposity (% of eWAT on total body weight) measured after 3 weeks (3w); 6 weeks (6w); 12 weeks (12w) and 16 weeks (16w) of a high-fat diet (HFD) or a control diet (CT) (n = 6/group). Data are presented as the mean ± SEM. Data are significantly different (*P* < 0.05) according to the unpaired two-tailed Student t-test. *indicates a significant difference versus CT (*P* < 0.05).

It is well established that HFD feeding promotes weight gain and adiposity leading to obesity. Here we found that HFD administration modestly, but significantly, increased body weight and adiposity after 3 and 6 weeks of dietary treatment. However, after this time point, we found a major effect of the diet on body weight gain, which rose up to ~45% after 12–16 weeks as compared to only ~15% after 6 weeks of treatment (Fig. 1A). Adiposity followed a similar pattern, with a dramatic increase at 12 weeks of age (~3.5 fold) as compared to control diet (CT) mice. This effect was even more pronounced after 16 weeks of HFD feeding (Fig. 1B). Collectively, these results indicate that the greatest impact on body weight gain and adiposity in mice occurs after 6 consecutive weeks of HFD administration.

### Markers of adipose tissue metabolism and inflammation were altered after HFD administration

**Figure 2:**
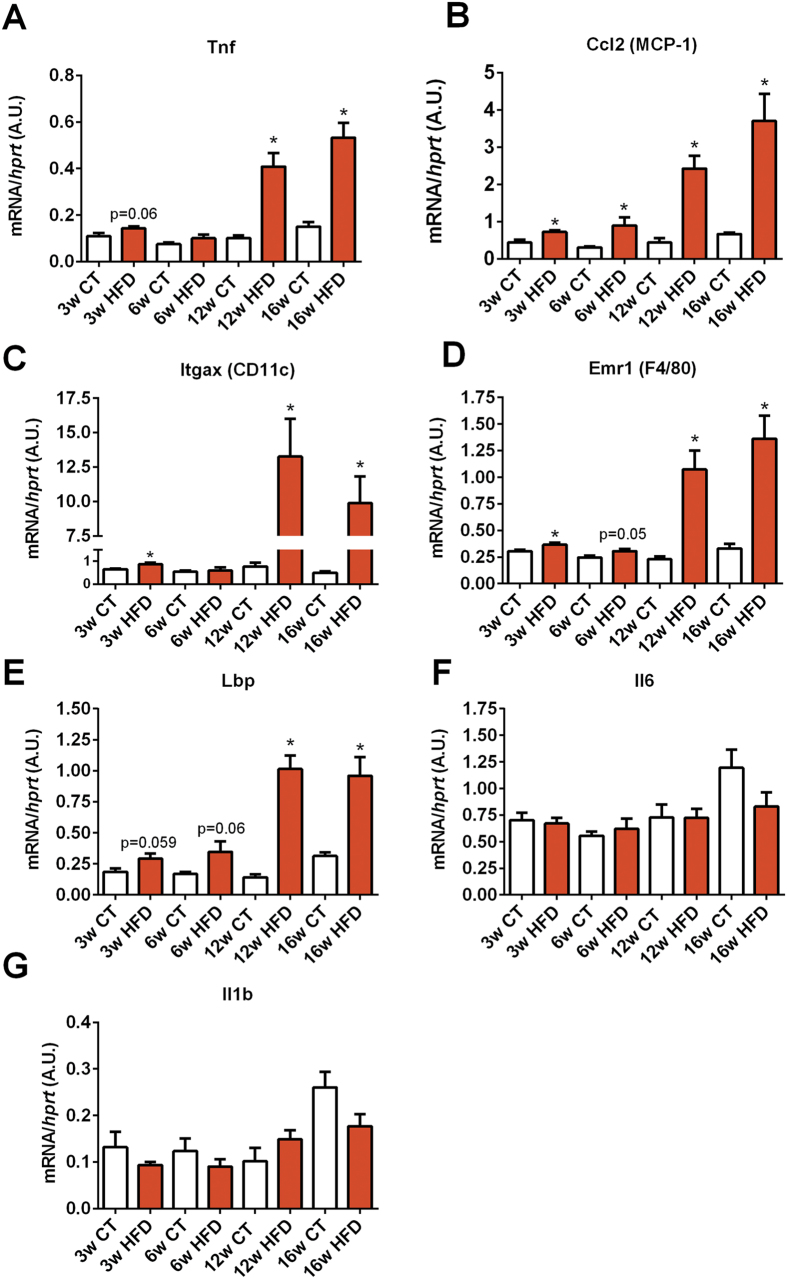
Time series evolution of markers of inflammation and macrophage infiltration in white adipose tissue following HFD administration. mRNA expression of (**A**) *Tnf* (encoding TNF-α), (**B**) *Ccl2* (encoding MCP-1), (**C**) *Itgax* encoding CD11c), (**D**) *Emr1* (encoding F4/80), (**E**) *Lbp* (encoding LBP), (**F**) *Il6* (encoding Il-6) and (**G**) *Il1b* (encoding IL-1β) measured in the adipose tissue after 3 weeks (3w); 6 weeks (6w); 12 weeks (12w) and 16 weeks (16w) of a high-fat diet (HFD) or a control diet (CT) (n = 6/group). Data are presented as the mean ± SEM. Data are significantly different (*P* < 0.05) according to the unpaired two-tailed Student t-test. *indicates a significant difference versus CT (*P* < 0.05).

We next assessed the repercussion of chronic HFD feeding on adipose tissue metabolism by measuring several markers of metabolic inflammation (*Tnf*, *Ccl2*, *Itgax*, *Emr1*, *Lbp*, *Il6*, *Il1*), fatty acid oxidation (*Acox1, Cpt1a, Acacb*), fat browning (*Cidea, Ppargc1a, Ppargc1b, Elovl3, Prdm16*), lipogenesis (*Dgat2*, *Fasn, Acaca*) and adipogenesis (*Pparg* and *Cebpa*) by quantitative PCR (qPCR). Macrophage infiltration markers (*Itgax* and *Emr1*) and recruitment of immune cells (*Ccl2*) were significantly increased as early as 3-weeks after the beginning of the HFD treatment (Fig. 2B–D), whereas markers of inflammation such as *Tnfa* and *Lbp* exhibited a trend to be increased but did not reach significance (Fig. 2A,E). After 12 weeks of HFD, all the aforementioned markers were strongly and significantly increased. Both *Il6* and *Il1* mRNA expression were not significantly affected by the treatment (Fig. 2F,G).

**Figure 3:**
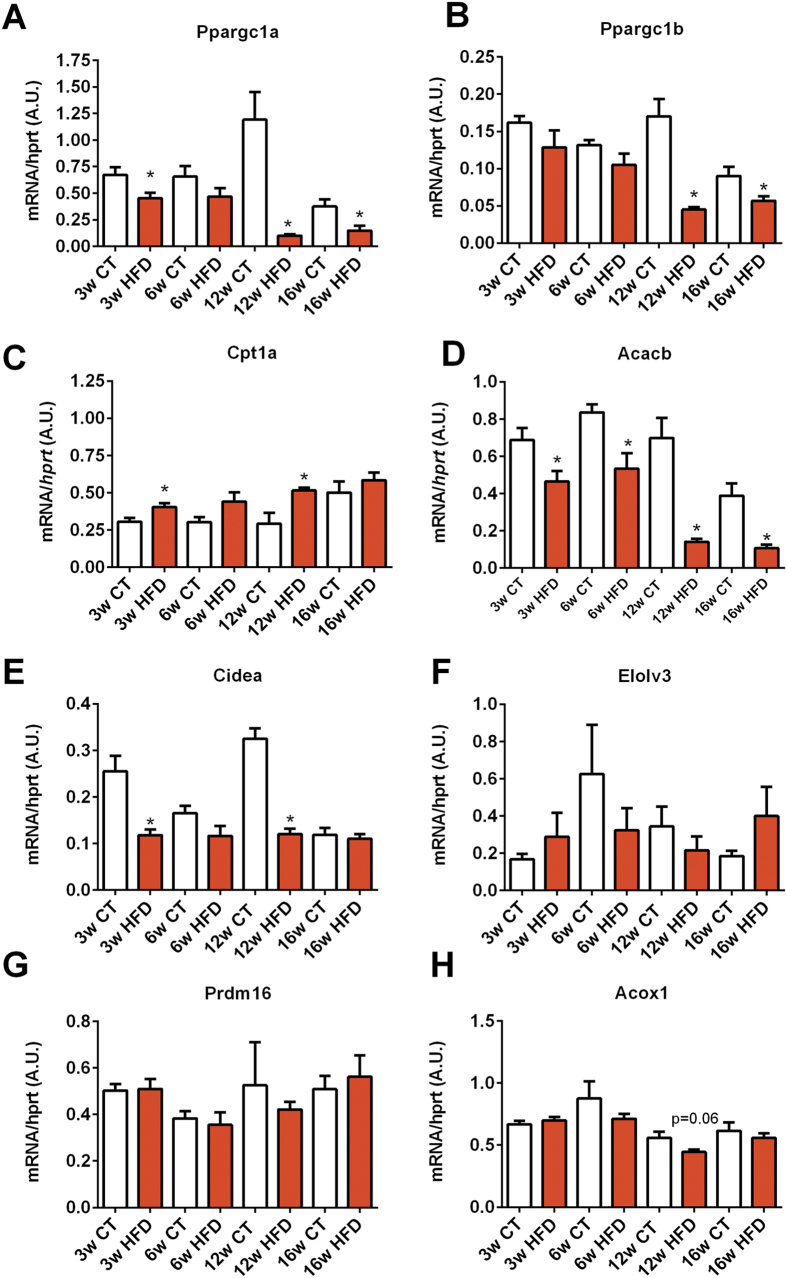
Time series evolution of markers of fatty acid oxidation and browning in white adipose tissue following HFD treatment. mRNA expression of (**A**) *Pppargc1a* (encoding PGC1-α), (**B**) *Pppargc1b* (encoding PGC1-β), (**C**) *Cpt1a* (encoding CPT-1a), (**D**) *Acacb* (encoding ACC2), (**E**) *Cidea* (encoding CIDEA), (**F**) *Elovl3* (encoding ELOVL3), (**G**) *Prdm16* (encoding PRDM16) and (**H**) *Acox1* (encoding ACOX1) measured in the adipose tissue after 3 weeks (3w); 6 weeks (6w); 12 weeks (12w) and 16 weeks (16w) of a high-fat diet (HFD) or a control diet (CT) (n = 6/group). Data are presented as the mean ± SEM. Data are significantly different (*P* < 0.05) according to the unpaired two-tailed Student t-test. *indicates a significant difference versus CT (*P* < 0.05).

Among the different markers of lipid metabolism involved in oxidation and browning program in white adipose tissue, we found that *Ppargc1a, Cidea,* and *Acacb* mRNA expression levels were significantly decreased after only 3 weeks of HFD (Fig. 3A,E,D), whereas other key markers of β-oxidation (*Acox1*) were not affected (Fig. 3H). *Ppargc1a, Ppargc1b and Acacb* transcript expression was dramatically decreased after 12 weeks of HFD and remained lower until the end of the dietary intervention (Fig. 3A–D). *Cpt1a* was slightly but significantly increased after 3, 6 and 12 weeks of treatment (Fig. 3C). *Elovl3 and Prdm16* mRNA expression levels were not affected by HFD throughout the experimental period.

**Figure 4:**
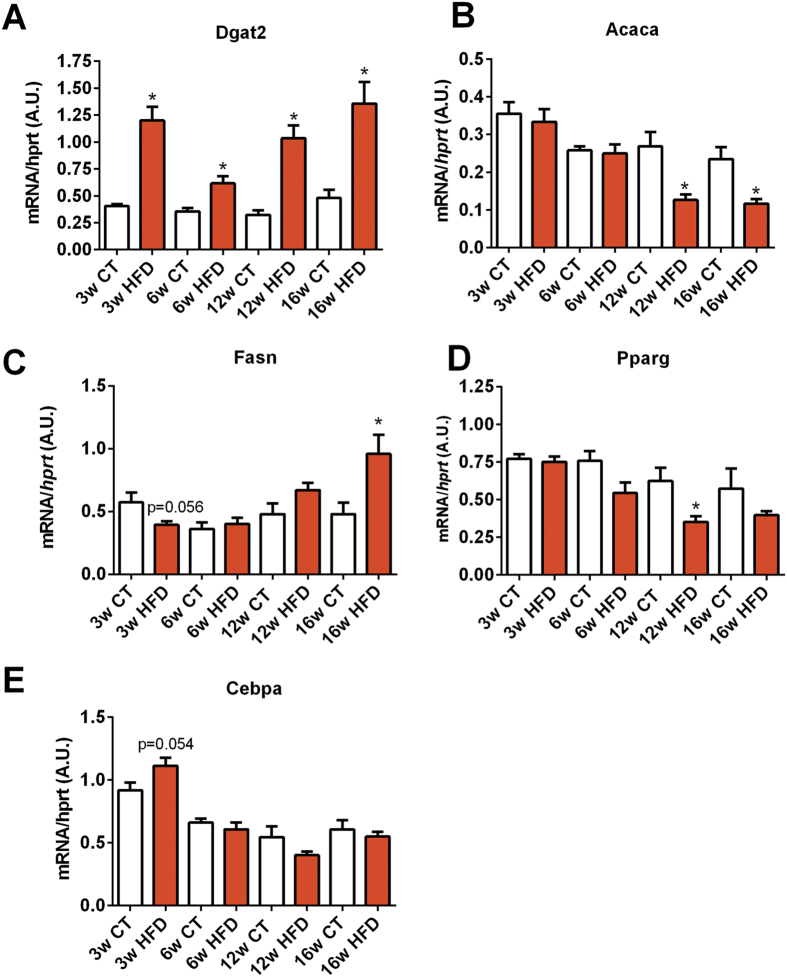
Time series evolution of markers of lipogenesis and adipogenesis in white adipose tissue following HFD treatment. mRNA expression of (**A**) *Dgat2* (encoding DGAT2), (**B**) *Acaca* (encoding ACC1), (**C**) *Fasn* (encoding FAS), (**D**) *Pparg* (encoding PPAR-γ) and (**E**) *Cebpa* (encoding CEBP-α) measured in the adipose tissue after 3 weeks (3w); 6 weeks (6w); 12 weeks (12w) and 16 weeks (16w) of a high-fat diet (HFD) or a control diet (CT) (n = 6/group). Data are presented as the mean ± SEM. Data are significantly different (*P* < 0.05) according to the unpaired two-tailed Student t-test. *indicates a significant difference versus CT (*P* < 0.05).

*Dgat2* expression, the enzyme that catalyzes the final step in triglyceride synthesis, was increased after 3 weeks of HFD and remained high during the overall period of time (Fig. 4A). Markers of lipogenesis were inversely affected by the HFD, while *Fasn* mRNA expression increased, *Acaca* transcript expression decreased after 16 weeks of dietary intervention (Fig. 4B,C). *Ppparg* and *Cebpa* mRNA expression levels, both markers of adipocyte differentiation, showed marginal changes over the course of the diet (Fig. 4D,E).

Collectively, the gene expression changes observed in white adipose tissue indicate that HFD administration causes profound changes in key genes implicated in inflammation, fatty acid oxidation and lipogenesis. Minimal effects were observed on genes mediating adipocyte browning and differentiation. In general, and in line with the physiological results, the most obvious alterations in gene expression were recorded after prolonged HFD treatments.

### Evolution of regulatory markers of food intake, diabetes and adiposity over 16 weeks of HFD

**Figure 5:**
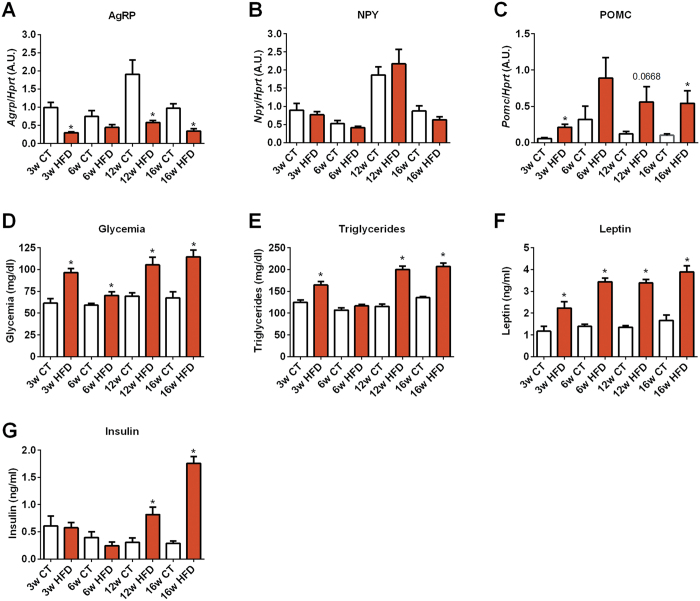
Time series evolution of orexigenic and anorexigenic markers in the hypothalamus and circulating levels of metabolic parameters following HFD treatment. mRNA expression of (**A**) *AgRP* (encoding AGRP), (**B**) *Npy* (encoding NPY), (**C**) *Pomc* (encoding POMC) measured in the hypothalamus,. Circulating levels of (**D**) glucose, (**E**) triglycerides, (**F**) leptin and (**G**) insulin measured in the serum of mice after 3 weeks (3w); 6 weeks (6w); 12 weeks (12w) and 16 weeks (16w) of a high-fat diet (HFD) or a control diet (CT) (n = 6/group). Data are presented as the mean ± SEM. Data are significantly different (*P* < 0.05) according to the unpaired two-tailed Student t-test. *indicates a significant difference versus CT (*P* < 0.05).

Defective energy balance is a major feature occurring during obesity. Energy balance is finely regulated by interactions between peripheral tissues, such as the intestine, and the central nervous system. In the hypothalamus, neuropeptide Y (NPY) and Agouti-related protein (AgRP) are orexigenic peptides, whereas proopiomelanocortin (POMC) precursor protein is cleaved into anorexigenic peptides. Interestingly, alterations in the expression of these neuropeptides have been extensively reported during HFD. Moreover, gut microbiota has recently been shown to be able to influence the expression of these neuropeptides in the hypothalamic arcuate nucleus of mice^24^. Therefore, we measured these parameters in our study. We found a decrease in the expression of orexigenic peptides such as AgRP during HFD, whereas the expression of anorexigenic peptides (POMC) were increased (at all studied time points) (Fig. 5A and C). Aging did not seem to impact on the expression of AgRP, NPY or POMC in the hypothalamus (Fig. 5A–C). We also found that markers of insulin resistance, such as fasted glycemia and insulinemia, progressively increased during prolonged HFD treatment. A similar trend was observed for both triglyceridemia and leptin levels (Fig. 5D–C).

### Evolution of selected gut bacteria during HFD feeding

**Figure 6:**
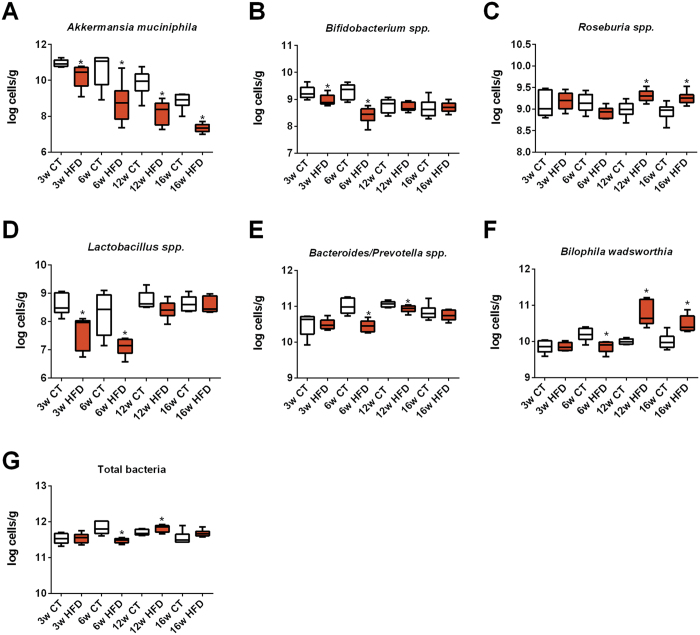
Time series evolution of specific gut bacteria following HFD treatment. Quantification of (**A**) *Akkermansia muciniphila,* (**B**) *Bifidobacterium* spp., (**C**) *Roseburia* spp. (**D**) *Lactobacillus* spp., (**E**) *Bacteroides/Prevotella* spp, (**F**) *Bilophila wadsworthia* and (**G**) total gut bacteria abundance after 3 weeks (3w); 6 weeks (6w); 12 weeks (12w) and 16 weeks (16w) of a high-fat diet (HFD) or a control diet (CT) and expressed as Log cells per g of caecal content (n = 6/group). Data are presented as the mean ± SEM. Data are significantly different (*P* < 0.05) according to the unpaired two-tailed Student t-test. *indicates a significant difference versus CT (*P* < 0.05).

Based on previous findings^8^,^14^,^15^,^16^,^17^,^18^, we measured the abundance of selected bacteria during the progress of HFD administration. We found that *Akkermansia muciniphila*, *Bifidobacterium* spp. and *Lactobacillus* spp. were significantly decreased after 3 and 6 weeks on HFD, although this decrease was transient for *Bifidobacterium* spp. and *Lactobacillus* spp. (Fig. 6A,B,D). The abundance of *Akkermansia muciniphila* decreased gradually to finally reach a level ~10,000 times lower than the initial one. Remarkably, under control diet, the levels of *Akkermansia muciniphila* progressively decreased by ~100 times as compared to young CT mice. Among the different bacteria the effect of aging was observed only for this taxon (Fig. 6A). We also observed that the abundance of *Roseburia* spp. and the abundance of *Bilophila wadsworthia* increased after 12 and 16 weeks of HFD treatment (Fig. 6C,F). Interestingly, the abundance of *Bilophila wadsworthia* was unaffected or even decreased during earlier time points of HFD (Fig. 6F). Both *Bacteroides/Prevotella* spp. and total bacteria were affected by the treatment at 6 and 12 weeks of age but were similar between groups at the end of the experimental period (Fig. 5E,F). Thus these data confirm that HFD feeding affects specific gut bacteria, and highlight that the abundance of *Akkermansia muciniphila* progressively decline with prolonged dietary treatment in CT mice, and that this effect is exacerbated upon HFD.

### Multifactorial analysis of adipose tissue markers and bacteria

**Figure 7:**
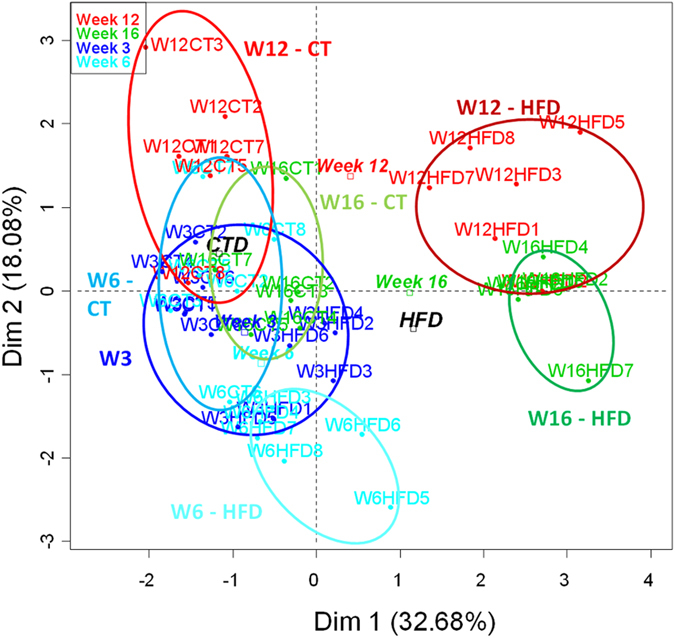
Individuals factor map. The individual factor map presents the repartition of the samples (dots) in the multifactorial analysis’ plane. Time and diet (squares) are presented as illustrative (inactive) qualitative factors. Samples are colored according to treatment’s duration. Circles regroup all samples from a specific diet for a single time point, except week 3 where all samples are grouped together.

In order to identify putative markers driving the modulation of metabolism in older animals and/or during HFD-feeding, we combined the bacterial taxa and metabolic parameters obtained at each time point in a multifactorial analysis. This multifactorial analysis takes into account all the different parameters measured in this study for each mouse, and allows us to determine if mice from the same group cluster together according to these metabolic parameters, thereby suggesting that altogether these parameters are key factors affected by age and/or HFD-feeding. The individual factor map shows the distribution of the samples according to the multifactorial analyses results (Fig. 7). All the samples from week 3 were clustered together, whereas samples from week 6 were separated along axis 2 according to the diet. We found that samples from weeks 12 and 16 were all separated along axis 1 according to the dietary treatment, thereby confirming the major effect of the diet on all the parameters. CT samples from weeks 3, 6 and 12 were all clustered together, while CT samples from week 16 were closer to the HFD samples from weeks 3 and 6. This observation suggests that older mice exhibit changes in the adipose tissue expression profile and microbiota composition similar to those observed during HFD at the early stage of obesity development (Fig. 7).

**Figure 8:**
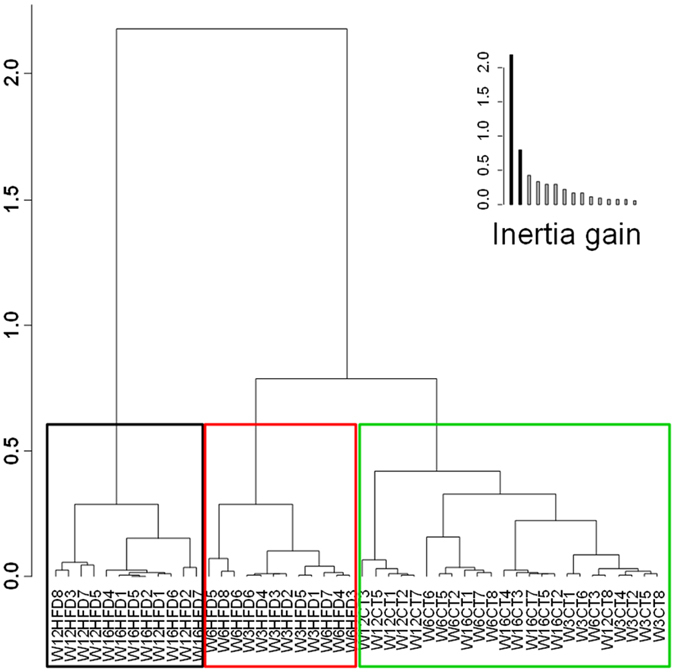
Hierarchical clustering. Hierarchical clustering of the samples on the principal components of the multifactorial analysis. This graph illustrates the similarities and the clustering between different samples. The smaller the distance linking samples is (resulting from the addition of horizontal distances in dark line), the more similar these samples are for the parameters measured.

The hierarchical clustering is another way to illustrate the similarity and the clustering between different samples. The smaller the distances (resulting from the addition of horizontal distances in dark line) linking samples are, the higher is the similarity for the parameters measured. The hierarchical clustering (Fig. 8) shows that HFD samples from weeks 12 and 16 grouped distant from the other samples. In addition, this graphical representation showed a robust clustering of CT samples from week 16 with HFD samples from week 6, emphasizing again putative similarities between HFD and aging. CT samples from weeks 6 (HFD) and 12 (CT)clustered together, reinforcing the results obtained with the individual factor map (Fig. 7).

### *Akkermansia muciniphila* strongly correlates with adipose tissue metabolic parameters

To further explore whether one specific bacterial taxa was correlated with host metabolism markers, we performed a multifactorial correlation circle and correlation map. This analysis is taking into account all the parameters measured in the study and determine if specific parameters are influencing or correlating with the others. It allows to determine the strength of the relation between the different parameters measured in the study. When the arrows are pointing in the same direction, this demonstrates that the parameters are positively correlated. If the arrows are pointing in opposite directions, the parameters are inversely correlated. When they are indicating different directions, this means that the parameters are not evolving in the same way and are likely not correlated. It is also important to mention that the length of the arrows is an indicator of the strength of the correlation.

**Figure 9:**
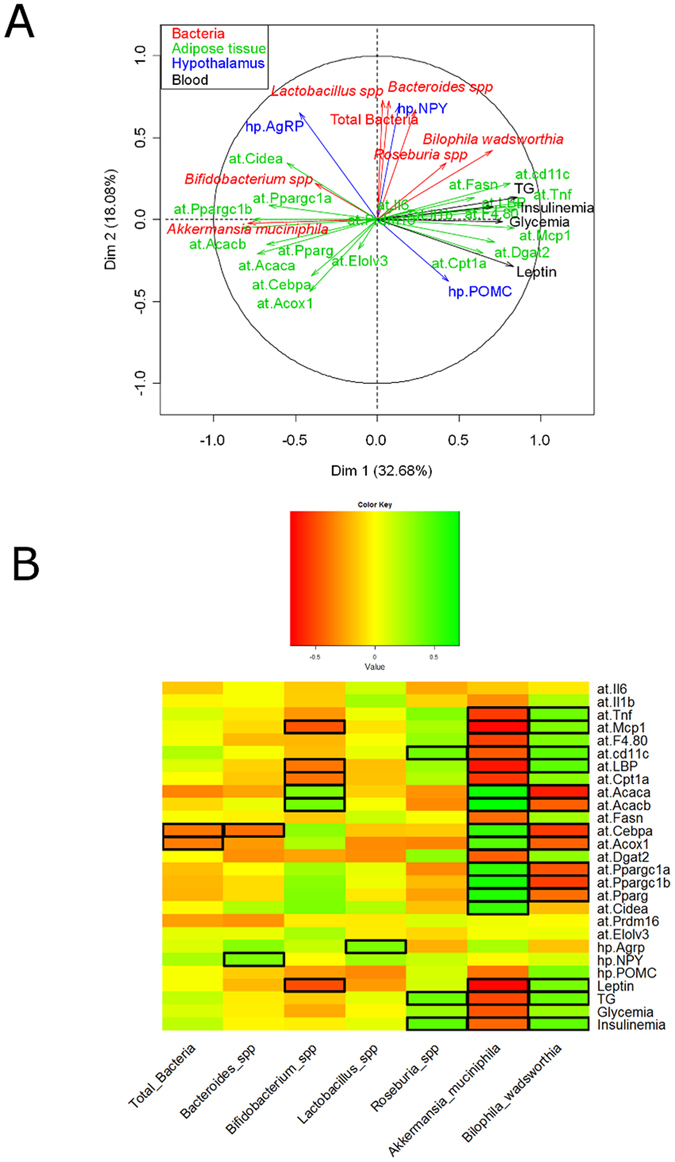
Correlation circle and heat map correlation showing associations between bacterial taxa and adipose tissue metabolic parameters. Multifactorial analysis (**A**) correlation map and (**B**) heat map of the Spearman r correlations between the bacterial genera and the metabolic parameters measured in the adipose tissue of mice after 3 weeks (3w); 6 weeks (6w); 12 weeks (12w) and 16 weeks (16w) of a high-fat diet (HFD) or a CT diet (CT) (n = 6/group). Data are presented as the mean ± SEM. Squared cells depict significance following the Spearman correlation and FDR correction for multiple comparisons *P < 0.05.

Surprisingly, we found that the levels of *Akkermansia muciniphila* were strongly and positively correlated with the levels of almost all the parameters involved in fatty acid oxidation and fat browning (Fig. 9A). Conversely, the levels of *Akkermansia muciniphila* were inversely associated with inflammatory markers, lipid synthesis, and several plasma markers of insulin resistance, cardiovascular risk and adiposity (Fig. 9A). *Bifidobacterium* spp. tended to exhibit a similar pattern as *Akkermansia muciniphila*, whereas the other taxa were not associated with adipose tissue metabolic and inflammatory parameters, except the pathobiont *Bilophila wadsworthia* showing a moderate but opposite trend to *Akkermansia muciniphila*. Interestingly, most of the inflammatory factors were strongly correlated with axis 1 on the right side (Fig. 9A), which mainly represents the direction of older mice samples (i.e., prolonged treatment time) as depicted in Fig. 7. *Roseburia* spp. tended to correlate moderately in this direction, while the other bacteria investigated were not associated with other parameters such as diet or aging (Figs 9A and 7). These observations were further reinforced using another mathematical model (Fig. 9B). *Akkermansia muciniphila* was significantly and inversely correlated with 5 out of 7 inflammatory markers, all circulating parameters (i.e., insulin, glucose, triglycerides and leptin) and positively correlated with 118 out of 13 genes involved in fatty oxidation, synthesis and browning, while *Bifidobacterium* spp. abundance was significantly and negatively correlated with 3 inflammatory markers, and leptin and positively correlated with only 2 gene involved in fatty oxidation. However, *Bilophila wadsworthia* points in an opposite direction than *Akkermansia muciniphila.* Although not fully correlated with the same parameters, *Bilophila wadsworthia* was positively associated with 4 out of 7 inflammatory markers, and negatively associated with 7 out of 13 genes involved in fatty oxidation, synthesis and browning and positively with 3 plasma parameters (i.e., insulin, triglycerides and leptin).

## Discussion

In the present study, we used targeted approaches to investigate the impact of HFD on specific gut bacteria abundance and metabolic parameters. Importantly, we focused our adipose tissue metabolic analysis on epididymal fat since this fat depot is an appropriate surrogate of whole-body white adipose tissue. This fat pad is particularly increased after HFD administration when compared to mesenteric fat^25^. In addition, a detailed analysis of the general inflammatory response upon HFD administration shows an early and more dramatic activation of the inflammatory program in epididymal adipose tissue in comparison with mesenteric^26^. Moreover, by using this epididymal fat, we could investigate several important markers of inflammation, adipogenesis and browning, whereas this latter parameter does not occur in another visceral fat pad such as mesenteric fat for instance. The taxa we have selected are the most commonly studied, and corresponded to bacteria that have been previously associated with HFD feeding by using several culture-dependent and independent strategies^1^,^7^,^8^,^9^,^13^,^14^,^15^,^17^,^19^,^27^,^28^,^29^,^30^.

We found that changes in specific gut bacteria occur progressively following HFD feeding. In particular, *Akkermansia muciniphila* was linearly affected by the dietary treatment. By using a number of strategies, such as multifactorial analysis, we found a robust association between this bacterium and particular changes in adipose tissue inflammation and metabolic parameters. Among the 27 parameters measured, 20 of them were significantly (positively or negatively) correlated with *Akkermansia muciniphila* (Fig. 9A,B). This suggests that this bacterium dialogue with host to maintain adequate adipose tissue metabolism and function, but this hypothesis warrants further direct investigation. Among all other bacteria measured in this study, *Bilophila wadsworthia* shows opposite trend with 14 of the 27 parameters significantly correlated with this bacterium. *Bifidobacterium* spp. has shown similar associations, but only few parameters (6 out of 20) were significantly correlated with this bacterium. Consistent with this, it has been reported a positive impact of *Bifidobacterium* spp. on metabolism during fat feeding^31^.

We have previously demonstrated lower levels of *Akkermansia muciniphila* in genetic obese (*ob/ob*) mice and mice fed a HFD for up to 5 weeks^1^,^14^. Other recent reports also found that the abundance of this bacterium is inversely associated with obesity and diabetes development following HFD administration^15^,^28^,^32^, thereby supporting our past and present findings. However, it is worth noting that recent studies have shown an increased abundance of *Akkermansia muciniphila* upon high fat and high carbohydrate (sucrose, maltodextrin, corn starch) diet feeding^33^,^34^, thus the mechanisms promoting the bloom of *Akkermansia muciniphila* in these reports warrants further investigation. We recently discovered that the composition of the fatty acids *per se* may also strongly contribute to the modulation of the abundance of *Akkermansia muciniphila*. We found that mice fed with a lard-enriched diet exhibited a significant decrease in *Akkermansia muciniphila*, whereas fish oil-enriched diet dramatically increased *Akkermansia muciniphila* in the gut and this effect was associated with a better control of the gut barrier function and lower adipose tissue inflammation, a phenomenon that can be transferred to germ-free recipient mice^35^.

Finally, another argument suggesting that *Akkermansia muciniphila* protects against body weight gain and adiposity development is based on studies showing that the simultaneous administration of HFD and *Akkermansia muciniphila* mitigated the impact of the diet in gut barrier dysfunction and body weight and fat mass gain^14^,^28^, although the underlying mechanisms are still unknown.

We found important variations of *Akkermansia muciniphila* quantity with for instance a 100-fold reduction over the period studied, however, the magnitude of the difference observed between inflammatory markers was not following the same trend. Although one would have expected a linear relationship, that is less *Akkermansia* corresponds to more inflammation, the present study as well as previous studies does not support the idea that such simple and strict linear relationship exists^14^,^28^,^36^,^37^. Thus, although we still do not know exactly the levels required to observe a shift between a healthy versus a pathological situation, it is likely that below a certain level (i.e., a threshold) of *Akkermansia muciniphila* gut barrier function and other physiological parameters (e.g., altered adipogenesis/browning) become altered. In accordance with this hypothesis, we have recently demonstrated in human that below a given quantity of *Akkermansia muciniphila*, subjects were less prone to positively respond to caloric restriction diet in terms of the improvement of inflammatory markers, insulin resistance and glycemia. Therefore, we do not rule out that the decrease in *Akkermansia muciniphila* observed in older mice in this study was not enough to reach the threshold level of *Akkermansia muciniphila* for inducing strong inflammatory markers and diabetes.

We have also revealed a link between *Akkermansia muciniphila* and age, since the intestinal levels of this bacterium also declined with age upon CT diet feeding. Interestingly, HFD strongly influenced adipose tissue profile and intestinal microbiota in a way that mimicked aging, or at least older mice. For example, young HFD fed mice (6 weeks of HFD) were clustered with older CT mice (12 weeks of diet). Moreover, older CT samples (16 weeks) displayed an intermediate state between young and long-term HFD samples. In humans, it has also been suggested that *Akkermansia muciniphila* progressively decreases in elderly subjects^38^, thereby corroborating our findings. Taken together, our results indicate that HFD administration is an aggravating factor of age, although we did not further investigate this question in mice followed throughout their entire lifespan.

Although previous studies have found a putative link between caloric intake and the abundance of *Akkermansia muciniphila*^39^,^40^, we did not found any relationship between this taxa and markers of hypothalamic control of food intake. Since HFD diet is associated with an increase in calorie intake, the decreased expression of orexigenic peptides (AgRP) during HFD coupled with the increased expression of anorexigenic peptides (POMC) (at all studied time points) in the hypothalamus strongly suggest a resistance to negative feedback usually associated with ingestion of caloric rich diet as reported in the literature^41^. The available literature shows a significant variability and inconsistencies in neuropeptide expression upon HFD administration. This is likely the consequence of the rodent strain used, the percentage of fat in the diet and the length of diet exposure. Nevertheless, our results are in accordance with the accepted general idea that HFD reduces orexigenic NPY and AgRP expression while increases POMC levels^41^.

In conclusion, our data show that diet-induced metabolic disorders in mice progressively develop with time reaching a maximal impact after 12 weeks of treatment. Moreover, the abundance of specific taxa, such as *Bifidobacterium* spp. and *Akkermansia muciniphila,* were strongly associated with markers of lipid metabolism and negatively associated with inflammation in adipose tissue, circulating glucose, leptin, triglycerides and insulin. Although both bacteria are early affected by the HFD treatment, *Akkermansia muciniphila* levels robustly decreased in a sustained manner until the end of the experimental period. This effect was also present during CT diet feeding, thereby suggesting the impact of age on this bacterium, since *Bilophila wadsworthia* that correlates with metabolic parameters was mainly affected after a prolonged dietary intervention (i.e., 12 to 16 weeks of HFD). Thus, although future studies are required to decipher the mechanisms underlying the rapid decline of *Akkermansia muciniphila* during HFD and aging, our data reinforce the putative beneficial impact of this bacterium on metabolism and the need of investigating its role in humans on the development of metabolic disorders associated with fat feeding and aging.

## Methods

### Mice and diets

Male age-matched C57BL/6J mice were purchased from Harlan Europe. Mice were maintained on a 12:12h light–dark cycle with free access to water and standard chow (Harlan Research Laboratories) or high-fat diet (45% Kcal from fat; Research Diets) for 3, 6, 12 and 16 consecutive weeks (starting at 6 weeks of age). All experimental groups were maintained and studied in parallel to minimize environmental effects. The whole study included n = 6 mice/diet/time point. Body weights were weekly determined. Mice were overnight fasted and culled by cervical dislocation. The entire epididydmal fat was carefully dissected and tissues (blood, fat, hypothalamus and caecal content) were rapidly snapped frozen in liquid nitrogen. To avoid potential variations between groups, tissues from mice fed with standard or HFD were alternately dissected. Mice from only one time-point were processed per day. Adiposity was measured by weighting the epididymal fat depot normalized by body weight. All *in vivo* studies were performed with approval of the University of Barcelona Ethics Committee, complying with current Spanish and European legislation.

### Quantitative PCR analysis of gene expression

Tissue mRNA isolated using Trizol (Invitrogen) following standard protocols. Total RNA was quantified using a NanoDrop spectrophotometer and cDNA generated using Taqman High-capacity Retrotranscription kit (Applied Biosystems). Quantitative PCR (qPCR) was performed as previously described^42^. Relative gene expression was calculated using the standard curve method. Proprietary Taqman Gene Expression assay FAM/TAMRA primers used (Applied Biosystems) were: Acetyl-CoA carboxylase 1 (*Acaca*; Mm01304257m1), Acetyl-CoA carboxylase 2 (*Acacb*; Mm01204671m1), Peroxisomal acyl-coenzyme A oxidase 1 (*Acox*; Mm01246834m1), chemokine (C-C motif) ligand 2 (*Ccl2*; Mm00441242m1), CCAAT/enhancer-binding protein alpha (*Cebpa*; Mm00514283s1), Cell death activator (*Cidea*; Mm00432554m1), carnitine palmitoyltransferase 1A (*Cpt1a*; Mm01231183m1), Diglyceride acyltransferase (*Dgat2*; Mm00499536m1), Fatty Acid Elongase 3 (*Elovl3*; Mm00468164m1), EGF-like module-containing mucin-like hormone receptor-like 1 (*Emr1*; Mm00802529m1), Fatty acid synthase (*Fasn;* Mm00662319m1), Interleukin-1 beta (*Il1b*; Mm00434228m1), Interleukin-6 (*Il6*; Mm00446190m1), Integrin alpha X (*Itgax*; Mm00498698m1), Lipopolysaccharide binding protein (*Lbp*; Mm00493139m1), Peroxisome proliferator-activated receptor gamma (*Pparg*; Mm01184322m1), Peroxisome proliferator-activated receptor gamma coactivator 1-alpha (*Ppargc1a*; Mm01208835m1), Peroxisome proliferator-activated receptor gamma coactivator 1-beta (*Ppargc1b*; Mm00504720m1), PR domain containing 16 (*Prdm16*; Mm00712556m1), tumor necrosis factor alpha (*Tnfa*; Mm00443258m1), Agouti-related peptide (*agrp*; Mm00475829g1), Neuropeptide Y (*npy*; Mm00445771m1) and pro-opiomelanocortin (*pom*c; Mm00435874m1). Hypoxanthine-guanine phosphoribosyltransferase (*Hprt*; Mm01545399m1) was used as housekeeping gene. mRNA levels were measured using the ABI Prism 7900 HT system (Applied Biosystems).

### Gut microbiota qPCR quantification

Metagenomic DNA was extracted from the caecal content using a QIAamp-DNA stool mini-kit (Qiagen, Hilden, Germany) according to the manufacturer’s instructions and the adapted procedure previously described^43^. The primers and probes used to detect the different bacteria were based on 16S rRNA gene sequences: Total Bacteria (Bacteria Universal) F-ACTCCTACGGGAGGCAGCAG, R-ATTACCGCGGCTGCTGG; *Bifidobacterium* spp. F-TCGCGTCYGGTGTGAAAG, *Bifidobacterium* spp. R-CCACATCCAGCRTCCAC, *Lactobacillus* spp. F-CCTTTCTAAGGAAGCGAAGGAT, and *Lactobacillus* spp. R-AATTCTCTTCTCGGTCGCTCTA; *Bacteroides-Prevotella* spp. F-CCTTTCTAAGGAGCGAAGGAT, *Bacteroides-Prevotella* spp. R- AATTCTCTTCTCGGTCGCTCTA; *Akkermansia muciniphila* F-CAGCACGTGAAGGTGGGGAC, *Akkermansia muciniphila* R-CCTTGCGGTTGGCTTCAGAT; *Roseburia* spp. F-AAGCGACGATCAGTAGCCGA, *Roseburia* spp. R-.TTCTTCTTCCCTGCTGATAGAG; *Bilophila wadsworthia* F-, ACCCTGGTAGTCCACGCTGT *Bilophila wadsworthia* R- TGAGTTCAGCCTTGCGACCG; Detection was achieved with a STEP one PLUS instrument and software (Applied Biosystems, Foster City, CA, USA) using MESA FAST qPCR MasterMix Plus for SYBR Assay (Eurogentec, Verviers, Belgium). Each assay was performed in duplicate in the same run. The cycle threshold of each sample was then compared to a standard curve (performed in triplicate) made by diluting genomic DNA (five-fold serial dilution) (BCCM/LMG, Ghent, Belgium and DSMZ, Braunshweig, Germany). The data were expressed as Log bacteria/g of cecal content as described in Everard *et al.*^14^.

### Statistical analysis

Data are expressed as means ± s.e.m. Differences between two groups were assessed using the unpaired two-tailed Student’s t-test. Data were analysed using GraphPad Prism version 5.00 for Windows (GraphPad Software, San Diego, CA, USA). Data related to the gut microbiota were analysed using JMP 8.0.1 (SAS Institute, Inc., Cary, NC) and R 3.0.2 (The R Foundation) with the RStudio 0.97.310 packages and gplots for the heatmap. Multifactorial analysis was performed using the FactoMineR 1.28 package for R (FactoMineR: an R package for multivariate analysis). The results were considered statistically significant at P < 0.05. False Discovery Rate (FDR) corrections for multiple comparisons were performed according to the Benjamini Hochberg procedure^44^.

## Additional Information

**How to cite this article**: Schneeberger, M. *et al.* *Akkermansia muciniphila* inversely correlates with the onset of inflammation, altered adipose tissue metabolism and metabolic disorders during obesity in mice. *Sci. Rep.* **5**, 16643; doi: 10.1038/srep16643 (2015).
